# Chasing the Origin of Viruses: Capsid-Forming Genes as a Life-Saving Preadaptation within a Community of Early Replicators

**DOI:** 10.1371/journal.pone.0126094

**Published:** 2015-05-08

**Authors:** Matti Jalasvuori, Sari Mattila, Ville Hoikkala

**Affiliations:** Centre of Excellence in Biological Interactions, Department of Biological and Environmental Science, University of Jyväskylä, Jyväskylä, Finland; University of Regensburg, GERMANY

## Abstract

Virus capsids mediate the transfer of viral genetic information from one cell to another, thus the origin of the first viruses arguably coincides with the origin of the viral capsid. Capsid genes are evolutionarily ancient and their emergence potentially predated even the origin of first free-living cells. But does the origin of the capsid coincide with the origin of viruses, or is it possible that capsid-like functionalities emerged before the appearance of true viral entities? We set to investigate this question by using a computational simulator comprising primitive replicators and replication parasites within a compartment matrix. We observe that systems with no horizontal gene transfer between compartments collapse due to the rapidly emerging replication parasites. However, introduction of capsid-like genes that induce the movement of randomly selected genes from one compartment to another rescues life by providing the non-parasitic replicators a mean to escape their current compartments before the emergence of replication parasites. Capsid-forming genes can mediate the establishment of a stable meta-population where parasites cause only local tragedies but cannot overtake the whole community. The long-term survival of replicators is dependent on the frequency of horizontal transfer events, as systems with either too much or too little genetic exchange are doomed to succumb to replication-parasites. This study provides a possible scenario for explaining the origin of viral capsids before the emergence of genuine viruses: in the absence of other means of horizontal gene transfer between compartments, evolution of capsid-like functionalities may have been necessary for early life to prevail.

## Introduction

Viruses are extremely abundant parasites of cellular organisms. Despite their prevalence and relative simplicity, it is unclear how the very first viruses emerged in this biosphere. Viruses are often stated to be polyphyletic, indicating that they have originated independently several times in the history of life. However, many of the different contemporary viruses are evolutionarily related despite their apparent polyphyletic origin [1], and in the past decade it has became increasingly more evident that the viruses of the most ancient organisms (i.e. bacteria and archaea) are also ancient [2, 3]. Although viruses do not share universally common genes such as those encoding ribosomal RNA in cellular organisms, viruses still do carry unique and evolutionarily deep-branching genes for which there are no cellular counterparts [2, 4]. These so-called hallmark genes are often essential for viruses to be able to maintain the viral life style, suggesting that they may have been evolving within viral genomes for billions of years already. Indeed, various studies hint that the origin of the viral ancestors occurred close to the stem of all genetic evolution [5–7].

One of the most common hallmark genes encodes capsid proteins [1, 2, 8, 9]. The capsid is the protective layer that encapsulates viral genetic material in the extracellular environment. The capsid also mediates the entrance of the viral genetic material into a new host cell upon infection. Due to these features, the capsid is essentially what makes a virus a virus: without its infectious particle (virion), a virus is unable to escape from the current host organism in an attempt to disseminate the parasitic genetic information to other potential hosts [10–12].

Virus capsids are composed of multiple copies of one or several types of major capsid proteins [9]. Structural comparisons of the major capsid proteins from different viruses have made it possible to posit viruses into ancient evolutionary lineages [1, 4, 13, 14]. These lineages often group together multiple families of viruses and thus, they have been argued to serve as a suitable way to organize viruses to even a higher taxonomic order than what has been officially recognized by the International Committee on Taxonomy of Viruses [13]. Viruses belonging to one structural lineage may contain members that infect hosts from different domains of life, suggesting that the viral ancestors were probably already infecting the last universal common ancestor of all cellular life [2, 15].

The evolutionary mechanisms resulting in the emergence of viruses are largely unknown despite of the fact that theoretical models have studied evolution of various types of parasites in primordial systems [16–18]. Yet, given that capsid is the characteristic feature of all viruses, the emergence of a ‘genuine’ virus therefore arguably coincides with the origin of viral capsids [5, 10, 12, 19]. This interpretation, however, leads to a question: does the origin of the capsid need to coincide with the origin of viruses? Or is it possible that the capsid was a preadaptation (or an exaptation) of primordial evolving communities that the first proto-viruses only subsequently adopted in order to excel in their parasitic strategy for survival?

Here, we set to investigate this problem. By doing so, we attempt to provide a potential explanation for the origin of capsid-forming genes before the origin of anything that could be accounted as a true virus. In other words, we study whether the formation of horizontal gene transfer inducing replicators (capsid-like genes) can be favorable (or even essential) for primordial life before the origin of parasitic viruses themselves. To achieve this, we utilize a model consisting of a matrix of abiotic compartments and simple ribozyme-like molecules that combine genetic information with enzymatic functions [8].

There are many different, although not mutually exclusive, models approaching and approximating the potential events that took place during the early evolution of life. These models have considered for example the origin of biochemistry [20, 21], the replication of early genetic information [22, 23], the evolution of enzyme specificity and cooperation [24] and the emergence of proto-cells from surface-bound replicators [25]. In all models, emerging forms of life need to be confined into compartments, proto-cells or on surfaces where reaction cycles, being those either or both replication of genetic information and/or chemical reactions, can be stably maintained [20, 25−29]. This is because repeated reaction cycles in rapidly diluting solutions, like in open ocean water, are difficult to explain.

Although compartmentalization or confinement in some other way may be necessary for early life, there is one particular problem that arises directly as a result of restricting antagonistic replicators within a common habitat. The emergence of parasitic agents (that exploit the resources but which do not contribute to their production) cripples the replication process [8, 26, 27, 30, 31]. Upon the emergence of a parasite, the system rapidly spirals into a cycle where all the ‘cooperative’ reactions produce resources that only the selfish and defective parasites capitalize on—and they do it to the extent that the replication cycles stop revolving. This evolutionary tendency of defective parasites to overtake and subsequently cause the collapse of closed systems has often been called as ‘the tragedy of the commons’, a term which we will also hereafter use [32, 33].

Different levels of selection provide a way to overcome the problems that parasites create within the system. Distributing replicators into multiple separate compartments allows natural selection to operate on the level of compartments instead of just genetic sequences [34]. Spatial structure makes it possible for those compartments that are free of parasites to maintain functional replication processes longer than others and thus their gene composition gets ‘group selected’ within the whole system [22]. In other words, a compartment, in which a parasite sequence arises, is eventually prone to die, but the other remaining compartments will still maintain replication cycles. In order for simulated life to survive over long periods of time, the models require some form of spatial dissemination of genes between compartments, on a surface or, alternatively, a mechanism that induces the division of proto-cells into new, separate systems [25, 26, 34, 35]. The horizontal movement and/or vertical redistribution of genetic information allows some of the regions to remain parasite-free, thus sustaining life even if the tragedies of the commons continuously occur in some parts of the system [36].

**Fig 1:**
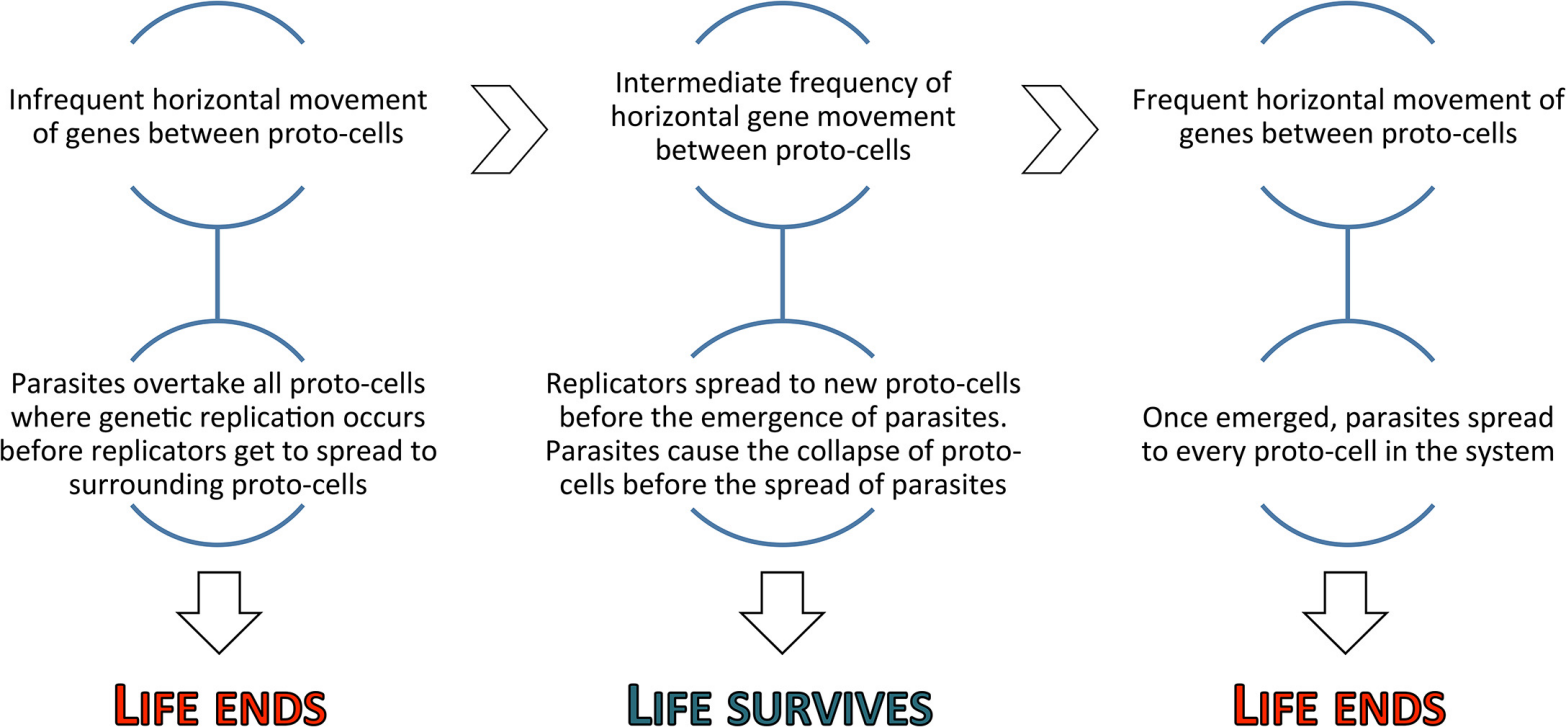
Both, too infrequent and too frequent horizontal gene transfer events lead to the collapse of the replication processes within the system. Intermediate transfer frequency, however, can cope with the presence of replication parasites.

Naturally, the tragedy of the commons is not avoidable by just any horizontal movement between compartments. Instead, the there is a precise threshold that sustains life within the metapopulation (Fig 1). Movement of genetic information between (static) compartments must not be too frequent, as the genetic exchange would serve only as a mean for parasites to spread within the system. On the other hand, too little horizontal movement results in the inevitable emergence of parasites in all of the compartments. However, with an intermediate frequency of horizontal transfer events, the parasites destroy themselves before getting the chance to spread to other compartments. Yet, non-parasitic replicators are likely to have a chance to get transferred across the system before the emergence of parasites, thus sustaining ‘healthy’ gene combinations in some of the compartments. Altogether, some form of genetic movement within a spatially structured community appears to be necessary for sustaining life and, indeed, many studies point towards an early life that was evolving horizontally rather than vertically [37, 38]. In some models, namely in surface bound metabolic systems, parasites may become destructive simply by becoming too common in its environment [25]. In this so-called *risk-of-common* mechanism, the different metabolic functions of the system decrease as any single gene becomes too abundant. Parasites eventually demolish stable metabolic cycles, thus causing local parasite extinctions. Naturally, even surface bound models require some spatial structuring and gene mobility to be able to form differing environments. Interestingly, Könnyu and colleagues have also shown that parasite sequences can explore sequence space and discover useful functions for the community [18]. Indeed, surface bound metabolic systems with parasites as a potential source of innovations present a possible evolutionary step towards the emergence of first functional proto-cells [25].

Recently, the early origin of actual viruses, not just sequences that parasitize replication reactions, has been incorporated to several discussions that attempt to tackle the evolutionary dynamics and the origin of different features in primordial living systems [2, 3, 39]. Also simpler virus-like replicators such as viroids that lack capsid-genes have been argued to be direct relics of the ancient RNA-world [40]. However, the origin of the very core of all viruses, the major capsid protein(s), has received less attention. Yet, as was recently noted by Krupovic and Forterre [10], arguably there can be no origin of viruses before there are the means to form a viral particle (i.e. transition from viroid-like replicators to viruses). Therefore, we here investigate whether the origin of a capsid-forming gene itself could be an evolutionarily favorable adaptation in a primordial compartment community due to their capability to induce horizontal gene transfer between compartments. Namely, we ask if primordial capsid-genes could protect the emerging system from collapsing to otherwise destructive parasites.

In this study, we demonstrate that capsid-forming genes are selected for in a simulated community of simple replicators where resource-exploiting parasites can emerge. Virus-like (but non-parasitic) structures generated via capsid-forming genes allow cooperative replicators to survive in the system. Our results suggest that genetically encoded viral capsids can be a prerequisite for life to prosper in the face of emerging replication parasites, thus providing a potential evolutionary pathway for explaining the origin of capsids before the emergence of genuine viruses. This can help us understand the ancient evolutionary processes that eventually lead to the origin of modern viral entities.

## Materials and Methods

### Description of the model

The model consists of genes that replicate within compartments arranged into a three-dimensional interconnected matrix. Genes are comparable to ribozymes: they both catalyze reactions and serve as a template for their own replication. Model also features resources, mutations and horizontal gene transfer (spontaneous or genetically encoded). In this system, dynamicity emerges from the iteration of the following computational steps: introduction of resources to each compartment; replication of genes; transfer of genes between compartments; and degradation of genes. Below, the model is described in detail.

### Genes and gene replication

**Fig 2:**
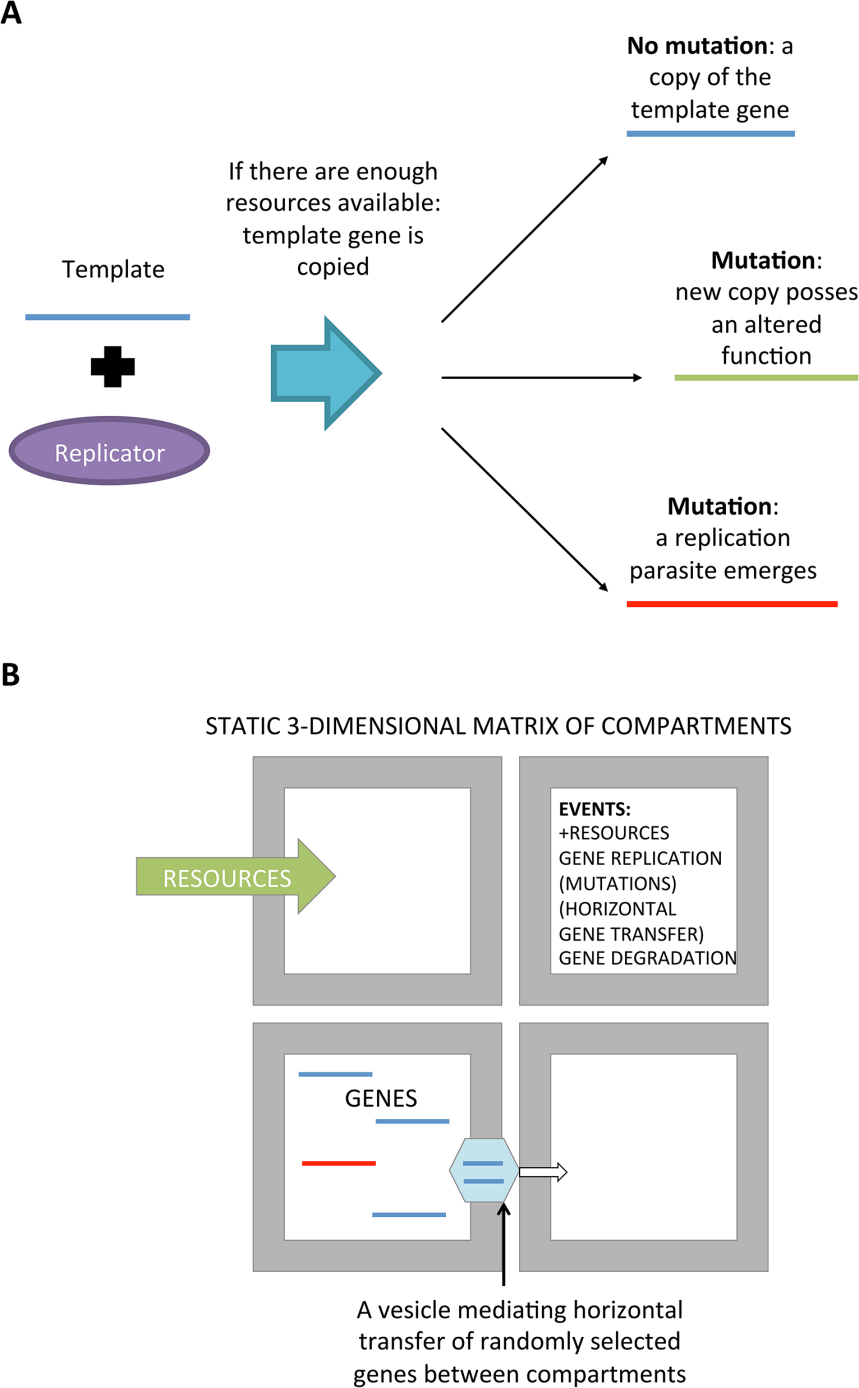
(A) In the developed model, replication of genes can induce mutations. These genetic changes can: change the type of the gene, render the gene useless (loss-of-function) or turn it into a replication parasite. (B) The model comprises a three dimensional matrix of static compartments in which all the simulated events take place. Horizontal movement within compartment is mediated via spontaneously and/or genetically induced (depending on the setup) forming of vesicles.

In this model, genes mediate different functions within the compartment while simultaneously serve as a template for their own replication. Template and coding strands are not modeled separately, that is, the template also posses the catalytic functionalities. The functions of different genes are listed in Table 1. Each gene-type may mutate into any other type if a replication error occurs during the duplication of the sequence by a replicase (Fig 2A). Gene replication can occur only if there are enough resources (adjustable by the user) for replication and replicases to catalyze the replication process.

**Table 1:** Different types of genes.

Gene type	Function	Adjustable parameters
Replicator	Replicates other genes within the same compartment	Probability by which a *replicase* finds a template within the compartment per an iteration of the simulation
Resource collector	Increases the amount of resources within the compartment	The amount of resources the *resource collector* adds to the compartment per an iteration of the simulation
Loss-of-function	A gene that has lost its earlier function mutation	
Replication parasite	Has an increased probability for attracting *replicators*	The probability by which *replication parasite* is being replicated by a *replicase*
Capsid-forming gene	Induces a horizontal movement of genes between compartments in the simulator	The number of *capsid-forming genes* required for mediating horizontal gene transfer

*Replicases* are genes that randomly select a template in its vicinity (compartment) for replication. Excluding replication parasites, all genes share an equal chance for being selected as a template for replication. The probability for a replicase to actually find any templates at all within the compartment can be adjusted. If there are enough resources available, single replicase can replicate one gene in an iteration of the simulation. In this study 10^5^ iterations of the simulation were ran for each setup, thus any replicase could catalyze the replication of maximum of 10^5^ replicators. *Capsid-forming genes* induce horizontal gene transfer events between compartments. *Loss-of-function* genes do nothing, but they can still be replicated if they get randomly picked as a template for a replicase. *Resource collectors* increase the amount of resources being introduced to the compartments (thus the number of replication events occurring within the compartment). By adjusting the levels of spontaneous resource inflow and the requirements for gene replication, it is possible to make the replication process to be dependent on the presence of different types of resource collectors. All genes act individually in this model: genes are physically separated entities and therefore there are no ‘genomes’ within the system.

### Mutations and replication parasites

Mutations occurring during the replication process randomly change the type of the copied gene into one of the other types (Fig 2A). Save for the probability for loss-of-function mutations as well as for the emergence of replication parasite to occur, changes between gene types takes place with the same probability. When any gene transforms into a replication parasite, the other catalytic functions of the gene are lost. This allows replication parasites to be identical with each other regardless of the original gene from which they have emerged.

The elevated replication probability of replication parasites indicates that, before any other gene replication events occur within the compartment, the replication parasites have a user-defined chance for becoming a template for the replicases. Only if a replicase does not select a parasite as a template, it proceeds to pick a random replicator as a template for replication (but it may still select a parasite by chance). Replication parasites are superior templates, but they have no catalytic functionalities.

### Compartments, gene degradation and resources

The compartments form a three dimensional matrix (Fig 2B). All dimensions are equal in size, that is, each dimension consists of an equal number of compartments. Therefore, a 4 x 4 x 4 matrix forms a system consisting of 64 compartments, and so on. Each compartment possesses its own resources, which are added to each compartment separately during every cycle of the simulation. Resource collectors can increase the amount of resource inflow. While replicators can become transferred between compartments, resources are compartment specific.

All genes have a steady degradation rate, indicating that in every simulation cycle there is a chance for a gene to disappear from the system. Degradation of a gene does not leave behind resources or ‘gene-fragments’. In practice, if there are no replication events occurring in the compartment, all genes will eventually be degraded: this is how replication parasites eventually cause their own destruction.

### Horizontal gene transfer

Genes can be transferred between compartments. Transfer occurs via the formation of a ‘vesicle’ that departs from one compartment and lands on another. The receiving compartment is selected randomly from all the possible compartments around the compartment from which the vesicle emerged. Those compartments that are on the edge of the matrix can also transfer vesicles to ‘outside of the system’, indicating that all genes within these vesicles are lost. Vesicles can form spontaneously or be actively induced by capsid-forming genes. Depending on the setup, either or both ways of horizontal gene transfer can occur within the system.

Spontaneously formed vesicles randomly enclose a user-defined number of genes. Induced vesicles must carry a user-defined number of capsid-forming genes (i.e. the genes that actually form the transfer capsule). The rest of the available space within the induced vesicles is filled with random genes present in the donor compartment. As the vesicle ‘fuses’ with the receiving compartment, all enclosed genes are introduced to the new environment. If there is no space available in the compartment, the genes will be degraded.

### Model setup for the study

**Fig 3:**
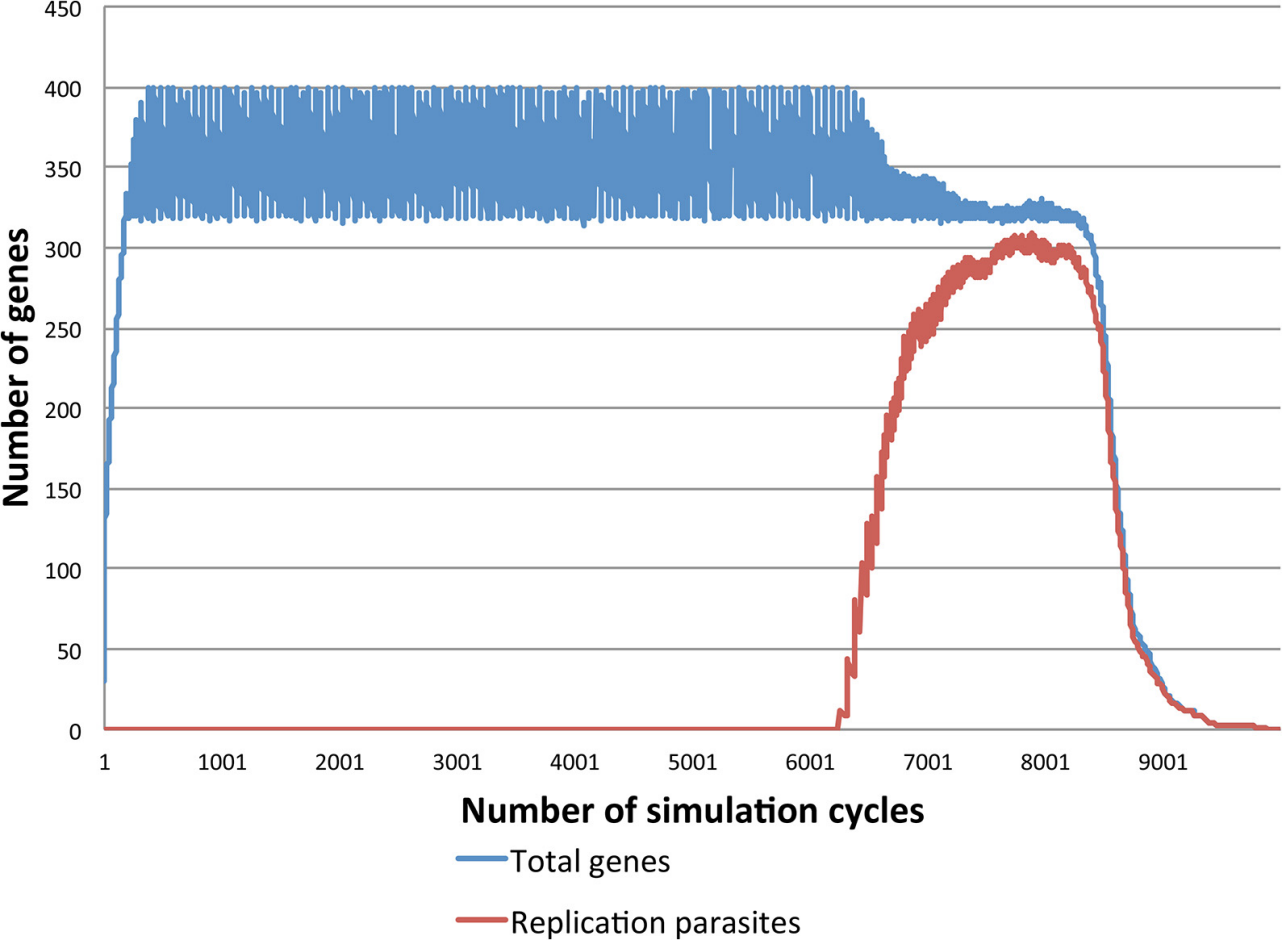
In the absence of horizontal gene transfer, the inevitable emergence of replication parasites leads to the tragedy of the commons: selfish replicators utilize all the resources without contributing anything to the system.

**Fig 4:**
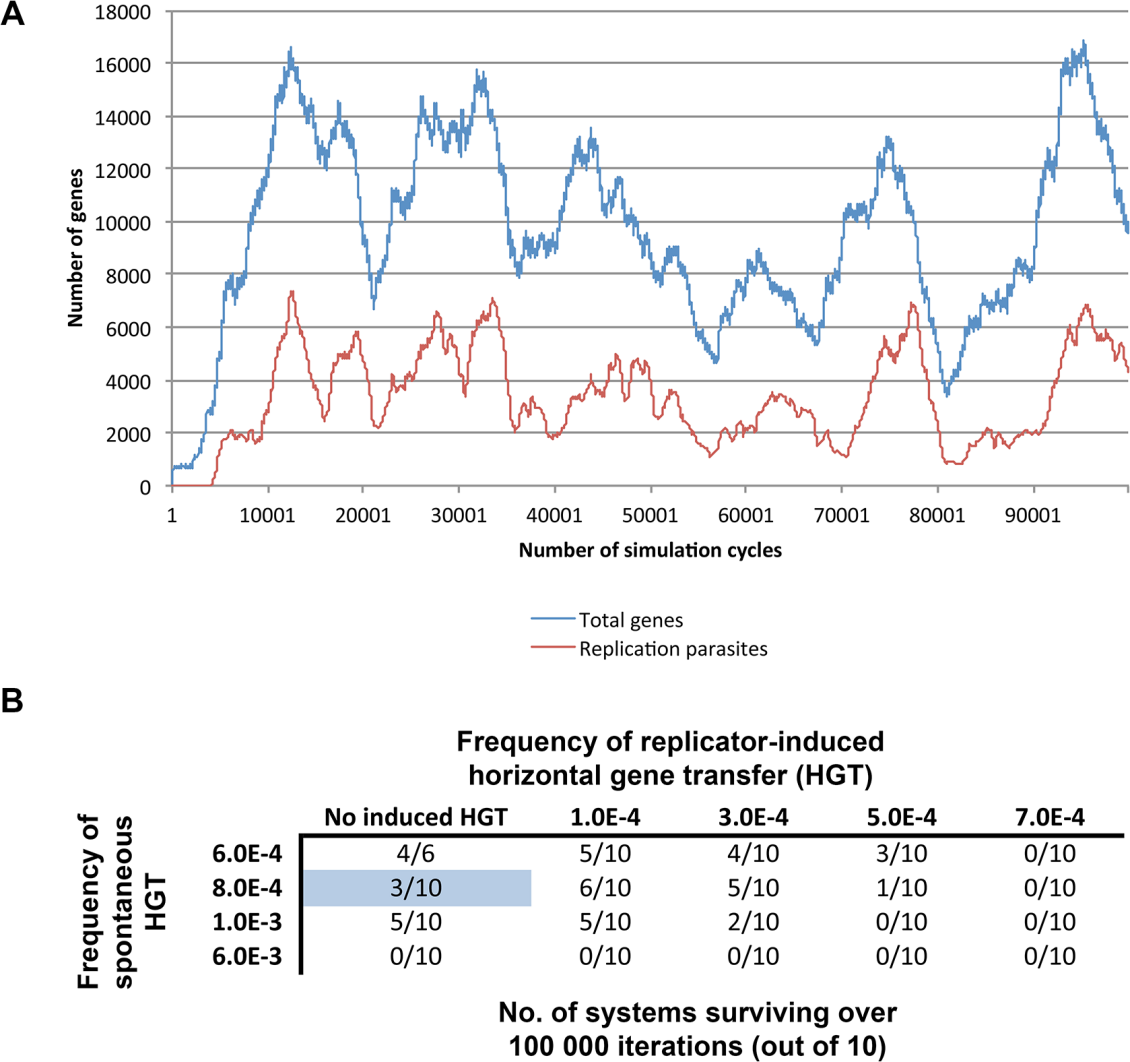
(A) Spontaneous horizontal gene transfer events allow replicators to spread within the system before the emergence of replication parasites. Size of the system was 5x5x5 (125) compartments and the spontaneous horizontal transfer rate was 8.0E-4 for each cell. (B) Simultaneous existence of induced and spontaneous horizontal gene transfer. The cells depict the number of simulations (out of 10) surviving over 100 000 iterations. Blue-cell indicates the conditions depicted in panel A. Other parameter-values are listed in Table 2.

As noted in the introduction, we set to investigate whether the emergence of genes mediating transfer of random set of genes between compartments can protect the system from collapsing due to the emerging parasites. In order to study this question, the system was set to meet the following conditions: First, in the absence of horizontal transfer of genetic information between compartment, the emergence of replication parasites must terminate life (as shown in Fig 3). Second, appropriate levels of random horizontal movement of genetic information between compartments must protect the system from collapsing to parasites (Fig 4A). Given these two conditions, it is possible to study whether genes that especially catalyze horizontal gene transfer events can be essential in the presence of emerging parasites. In other words, we ask whether the spontaneous transfer events may be replaced with genetic information that induces the horizontal movement of genes between compartments.

The exact parameter values used are described in Table 2. The simulator is freely available at www.jyu.fi/coe-virus/primordialevo (requires Java 6 or higher to run).

**Table 2:** Values of the attributes used in simulations.

Attribute	Value
Compartments in the system	Varies
Chance for a replicase to find a template	1.0
Resources needed for the replication of a gene	10
Number of capsid-genes needed for induced vesicle formation	15
Chance for induced vesicle formation when gene requirements are met	Varies
How many genes are enclosed within the induced vesicles	20
Chance for a random vesicle formation to occur	Varies
Gene degradation rate	0.004
Chance of a mutation to occur per gene replication event	0.02
Chance of a mutation to lead into the loss-of-function	0.9
Maximum number of genes per compartment	Varies
Chance for a replication parasite to emerge per gene replication event	Varies
Chance for a replication parasite to be selected as a template	0.5
Amount of resources collected per a collector gene	10
Maximum resources in a compartment	1000
Amount of initial resources in compartments	1000
Passive inflow of resources to compartments per simulation cycle	20
Initial number of replicases	10
Initial number of capsid-forming genes	10

## Results

Simulated systems encompassing replicases and other replicators within a compartment matrix that has no potential for horizontal gene transfer rapidly collapse due to the emergence of replication parasites (Fig 3). Selection favors those sequences with the highest probability for being replicated (i.e. replication parasites), and therefore, all the resources within the compartment are wasted on replicating defective parasites. However, this outcome changes when the system is set to have a possibility for spontaneous (i.e. non-genetically induced) movement of genes between compartments. With suitable compartment-to-compartment transfer frequencies, the system is able to remain alive significantly longer (Fig 4A). In these systems, the emergence of replication-parasites cause limited tragedies, because the parasite-epidemic is unable to spread to other compartments before the parasites themselves are naturally degraded from the parasitized compartments. A fraction of the compartments will remain free of parasites and these cells serve as the ‘healthy’ supply of replicases and other replicators that will eventually spread to other compartments within the system. Furthermore, we investigated whether both induced and spontaneous horizontal gene transfer can co-exists within a single system (Fig 4B). As expected, the combined frequency of horizontal gene transfer events determines whether or not the system can remain alive.

**Fig 5:**
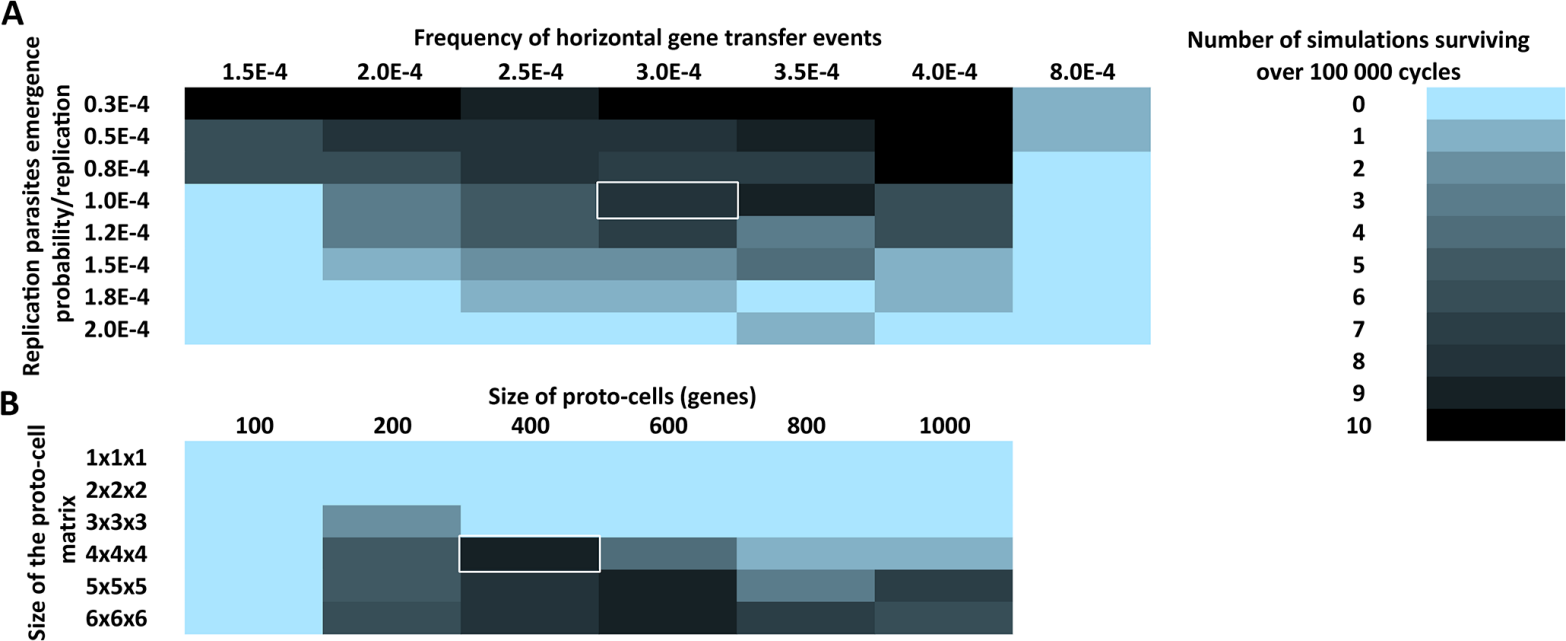
Survival of life over 100 000 simulation cycles depending on (A) the frequency of horizontal transfer events and the probability for the emergence of replication parasites, or (B) the size of the compartments matrix and the size of each compartment within the matrix. All simulations were run ten times and the average number of simulations surviving over 100 000 cycles is presented. The cell with white borders in (A) has the attribute values used in (B), and vice versa.

Finally, we conducted series of simulations with differing parameters to investigate whether the existence of genes that specifically induce compartment-to-compartment transfer events can altogether replace spontaneous horizontal gene transfer as a mean to protect the system from collapsing upon the emergence of replication-parasites (Fig 5). In these simulations, there was no spontaneous movement of replicators between compartments, thus all events of intercellular mobility were genetically induced. All simulations were run either 100 000 cycles or as long as there were genes left in the system, and each simulation setup were repeated ten times in order to rule out stochastic randomness.

The relationship between the size of the system and the survivability of life was investigated (Fig 5A). We observe that systems composed of just a handful of compartments are unable to survive. However, simulations consisting of a 4x4x4 compartment matrix (64 cells) were already able to bear with the emerging parasites. Yet, compartments that had more space for genes selected against survivability. This was due to the increased time it took for all the parasites to become degraded from the compartment after the local tragedy of the commons. Thus, as long as there remained functional replicator-parasites within a compartment, the incoming non-parasitic replicators were unable to start a gene community where cooperative (and non-parasitic) templates could be stably maintained via continuous replication cycles. Only after the complete degradation of parasites it is possible for replicators to successfully resettle into a compartment.

We also investigated how variation in horizontal gene transfer rates and parasite emergence frequencies affect the survivability of life. The horizontal transfer rate refers to the chance at which a ‘gene transferring vesicle’ will be formed when the compartment contains a sufficient number of copies of the capsid-forming genes (15 in this case). The parasite emergence frequency refers to the chance at which a replication-parasite is formed during the replication of any gene. Similar to spontaneous transfer events, too low and too high number of transfer events result in the demise of the whole system (Fig 5B). Moreover, as was expected, the higher the probability for parasites to emerge, the less likely was the system to survive over 100 000 cycles.

## Discussion

Although the ancient origin of viruses is a plausible scenario in the light of current evidence [41], the actual mechanisms leading to the emergence of sophisticated parasites that are both able to exploit cellular resources and to mediate their own transfer into a new host cell is far from being understood. Here, we demonstrate that horizontal gene transfer activity induced by ‘capsid-forming genes’ can protect a primitive, spatially structured community from collapsing even if parasitic templates continuously inhabit the system. Therefore, emergence of a replicator that possess essentially the same function with viral capsid can resolve the tragedy of the commons and, in the absence of other means of horizontal gene transfer, may be a requisite for the survival of life. Importantly, the horizontal gene transfer as induced by these capsids in our simulation does not discriminate between the genes that get transferred, thus the ‘capsid gene’ is selected only for its precise function and not for its potential to spread parasitic sequences to new host cells. To the best of our knowledge, this notion provides, for the first time, an evolutionary explanation for the emergence of the constitutive factor of all viruses—the capsid (or capsid-like genetic functionality)—before the origin of genuine viruses themselves. However, it must be noted that the ‘capsid’ studied in our simulation must not be treated as the precursor of any particular capsid protein currently existing in the biosphere. Also, we explicitly avoided the speculation on the actual nature of the ‘capsid’ as we did not want to confine our model to any particular origin-of-life scenario. Moreover, there are also other non-viral cell-encoded systems that transfer genetic material (along with other biomolecules) between cells. In animals, for example, exosomes can transfer nucleic acids between cells while clathrin encoded vesicles bud from the cell-surface to mediate endocytosis of various types of molecules and entities (including viruses) [42, 43]. Also bacteria and archaea can encode viral membrane-vesicles that enclose chromosomal and extrachromosomal DNA [44, 45]. Therefore, while we have focused in our discussion on the origin of potential precursor of viral capsids, it is certainly possible that the very same evolutionary tendencies may have also, or possibly only, contributed to the origin of other horizontal-transfer mechanisms.

Different stages of early life contained varying levels of genetic complexity. The selection pressures and physical constraints along with ecological factors were also different at different evolutionary stages. It is possible that life emerged in a relatively static matrix of compartments [28] or on a surface [25] where metabolic reactions and the replication of genetic templates could be maintained. Sooner or later, template competition favors the emergence of replication-parasites. At some point lipid layers or other less-permeable membranes formed to surround the emerging proto-cells during the life’s evolutionary trajectory towards independently replicating cells [15, 27, 28, 35]. Therefore, based on the results, it is possible that there was a stage during the evolution of early life when the invention of capsid-forming genes may have been a life-saving adaptation. It is also possible for induced gene transfer to co-exist with spontaneously occurring transfer events. Nevertheless, capsid-forming genes may have served as the stepping-stone towards the establishment of actual viruses, and thus, possibly, for the origin of the speculated virus-world that preceded the origin of modern bacterial and archaeal cells [2]. Paradoxically, the first capsid-forming genes may have initially protected life against parasites while also paving the way for the emergence of most successful parasites (in terms of abundance) in the history of life on earth [19].

Naturally, our results are obtained from simulations with a limited complexity, and thus, as with all evolutionary models, caution needs to be exercised when they are applied to the real world or to actual events that took place on early earth. For example, we did not take into account the possibility that capsid genes may evolve to become better or worse at facilitating the transfer of genes, and thus, as has been shown for spatially structured systems of higher organisms, evolution may modify the transfer frequencies to become less optimal for the survival of the community [46]. On the other hand, the copying fidelity of the replicases should increase when the dispersal rate decreases, thus slowing down the frequency by which new parasites emerge [23]. Also, different defensive measures to neutralize parasite sequences may have evolved early [15, 47]. Therefore, the optimal strategies and compositions of early living systems were likely to fluctuate over time and space. Thus, relatively simple modeling studies cannot precisely capture the stochastic and historic events that were responsible for the origin of viruses. Yet, models can provide evolutionarily feasible scaffolds, in which the emergence of new entities, such as viruses, seems less mysterious.
